# Acoustics-Actuated Microrobots

**DOI:** 10.3390/mi13030481

**Published:** 2022-03-20

**Authors:** Yaxuan Xiao, Jinhua Zhang, Bin Fang, Xiong Zhao, Nanjing Hao

**Affiliations:** 1 Key Laboratory of Education Ministry for Modern Design and Rotor-Bearing System, Xi’an Jiaotong University, 28 Xianning West Road, Xi’an 710049, China; xyx0805@stu.xjtu.edu.cn (Y.X.); binfang@mail.xjtu.edu.cn (B.F.); 2 Laboratory of Microscale Green Chemical Process Intensification, School of Chemical Engineering and Technology, Xi’an Jiaotong University, 28 Xianning West Road, Xi’an 710049, China; x_zhao@xjtu.edu.cn

**Keywords:** acoustics, microrobots, 3D printing, photolithography, microrotor

## Abstract

Microrobots can operate in tiny areas that traditional bulk robots cannot reach. The combination of acoustic actuation with microrobots extensively expands the application areas of microrobots due to their desirable miniaturization, flexibility, and biocompatibility features. Herein, an overview of the research and development of acoustics-actuated microrobots is provided. We first introduce the currently established manufacturing methods (3D printing and photolithography). Then, according to their different working principles, we divide acoustics-actuated microrobots into three categories including bubble propulsion, sharp-edge propulsion, and in-situ microrotor. Next, we summarize their established applications from targeted drug delivery to microfluidics operation to microsurgery. Finally, we illustrate current challenges and future perspectives to guide research in this field. This work not only gives a comprehensive overview of the latest technology of acoustics-actuated microrobots, but also provides an in-depth understanding of acoustic actuation for inspiring the next generation of advanced robotic devices.

## 1. Introduction

As early as 1959, famous physicist Richard Feynman proposed in his speech: there is plenty of room at the bottom [1]. In his vision, these micro/nanoscale objects could be used for benefiting human beings in many kinds of situations. Since then, with the maturity of micro/nanotechnology and mechanical manufacturing, Feynman’s vision has gradually become a reality. With the development of science and technology, there is an urgent need for people to develop delicate techniques that work on tiny objects or their postures, which can be undertaken by microrobots. Microrobots mostly reflect natural creatures, mimicking the appearance and movement of insects and small animals. According to their different forms of motions, microrobots can be divided into crawling robots, flying robots, and swimming robots. To date, these microrobots have received considerable attention in the fields of environmental monitoring, device flaw detection, military, and many other fields due to their small size, light weight, high flexibility, and diverse motion modes. However, due to the limitation of methodologies and difficulties in technological breakthroughs, many studies are still in the exploratory research phase.

For the actuation of microrobots, the motor cannot be used as a power source like ordinary robots due to the size mismatch. Therefore, researchers have developed many different actuation methods, such as magnetic actuation, acoustic actuation, biological actuation, and chemical actuation. Magnetic actuation has the advantages of strong penetration, remote control, and non-destructive actuation for living organisms, but the safety of high-strength magnetic fields needs to be carefully considered, especially for biomedical applications. Biological actuation can be generally classified into bacteria-based actuation and eukaryotic cell-based actuation, but the organisms often show low structural flexibility and have a poor lifespan. Chemical actuation usually relies on the energy generated by chemical reactions to power the microrobots. However, deficiencies are obvious such as the high risk of in vivo cross-reactivity, insufficient propulsion accuracy, short action time, and lack of instantaneous feedback. Acoustics can be regarded as a robust and reliable source to remotely manipulate microrobots. Comparatively, acoustic actuation offers many typical advantages in terms of strong penetration, high flexibility, and good biocompatibility, making it highly desirable for many emerging fields [2,3]. Table 1 shows the comparison of different actuation methods. In recent years, acoustics-actuated microrobots have gained considerable attention due to their promising potential use in diverse fields [4,5,6,7,8,9,10,11]. With the aid of acoustic actuation, many critical tasks can be performed such as targeted drug delivery [12,13,14], particle separation [15,16,17], mixing [18], pumping [19,20,21,22], self-assembly [23,24,25], microsurgery [26], microfluidic operation [27,28,29], and chemical analysis [30].

**Figure 1:**
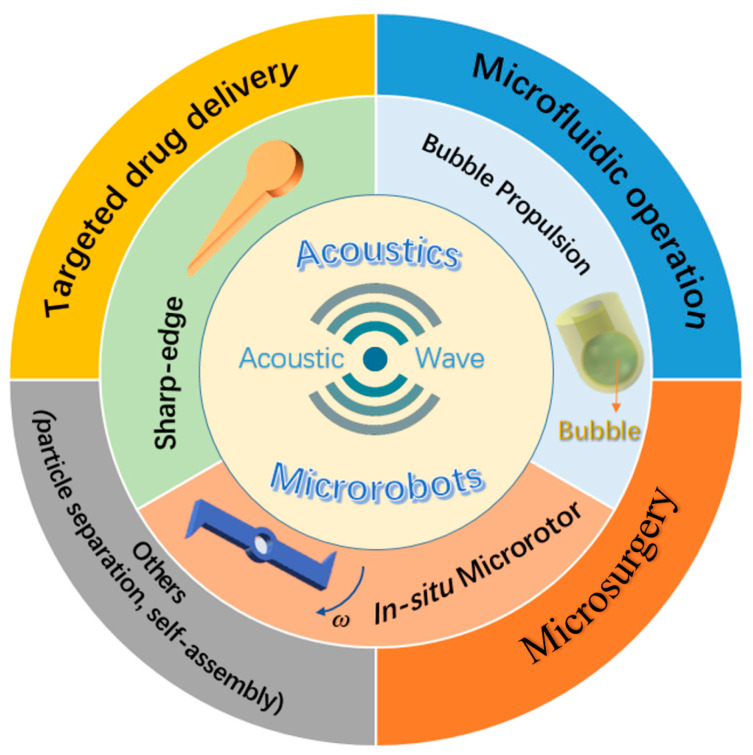
Classifications and applications of acoustics-actuated microrobots.

Herein, this review article provides a comprehensive overview of acoustics-actuated microrobots. We start by outlining common manufacturing methods of microrobots, followed by introducing different types of acoustics-actuated microrobots and application fields of acoustics-actuated microrobots (Figure 1). Finally, the current challenges and future research directions are presented. Owing to the unique merits of acoustic actuation, microrobots are expected to not only greatly improve the application outcome, but also significantly broaden the application range.

## 2. The Manufacturing of Microrobots

When applying acoustics to microrobots, the properties of materials play significant roles in their performance. To date, different kinds of materials such as polyethylene, Teflon, and Parylene have been employed to create microrobot devices [31]. In this section, we will present several typical methods for microrobot development, including 3D printing and photolithography.

### 2.1. 3D Printing

Three-dimensional printing represents one of the rapid prototyping technologies. It is based on digital model files, using powdered metal, plastic, or other bondable materials to construct the object through layer-by-layer printing. Three-dimensional printing is widely used in the fields of biomedicine, microfluidic devices, micro-/nanooptics, micro-/nanosensors, and biochips [32,33,34,35,36,37]. Due to its superior features in terms of simple equipment, low cost, high efficiency, and direct forming, 3D printing exhibited promising performance for use in the manufacturing of complex micro-/nanodevices [38,39,40].

**Figure 2:**
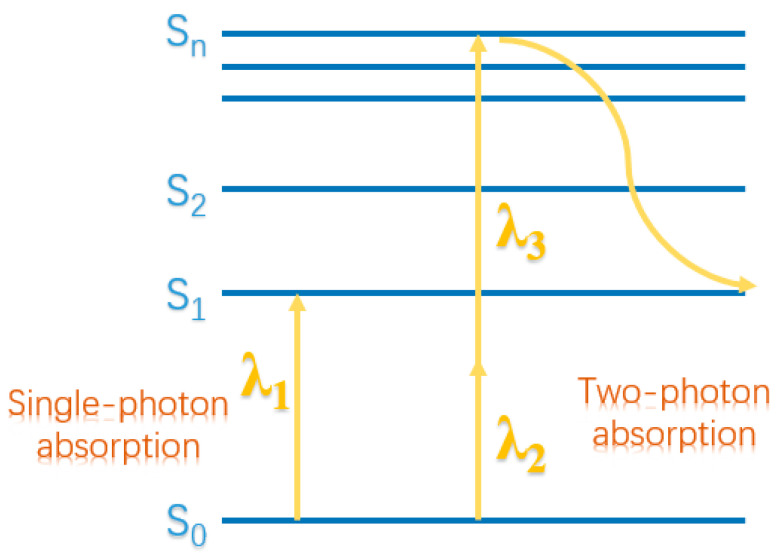
The difference between single-photon absorption and two-photon absorption.

Two-photon polymerization is a typical 3D printing technology that relies on the photopolymerization process initiated by a substance after two-photon absorption. The difference between two-photon and single-photon absorption is that when a substance undergoes single-photon absorption, it only needs to absorb a photon with a wavelength of λ_1_ to go from the initial state S_0_ to the excited state S_1_; while two-photon absorption requires a substance to simultaneously absorb two photons with the same or different wavelengths to reach the excited states S_1_, S_2_, S_n_, and then undergo a non-radiative transition to S_1_ (Figure 2). Generally, a substance can simultaneously absorb two photons at the focal point of the two beams of light to initiate the polymerization reaction. Therefore, two-photon polymerization usually occurs in places where the light is strong enough [41]. Two-photon polymerization has the advantages of good spatial selectivity and strong material penetration, making it desirable for the manufacturing of microrobots. The Photonic Professional GT from Nanoscribe GmbH has been successfully developed for printing polymer microrotors [42]. Two-photon polymerization occurs at the focal point of the beam. The beam position can be quickly scanned by a current mirror in the XY plane, while its Z position is determined by a piezoelectric translational platform, thus enabling cross-linking of the resist in all three dimensions. According to this way, two-photon polymerization lithography can be employed to fabricate diverse microrotors for many specific applications.

### 2.2. Photolithography

**Figure 3:**
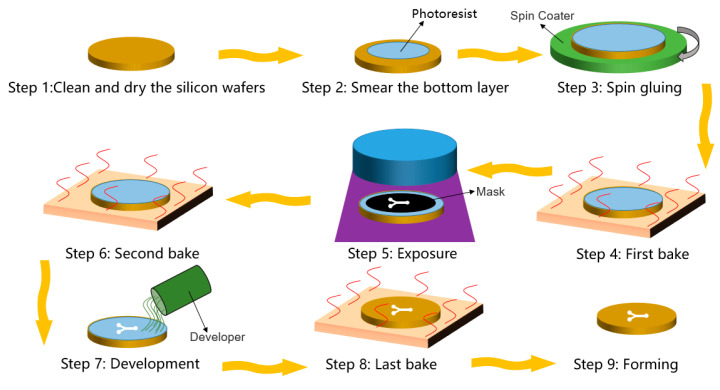
General steps of photolithography.

Photolithography refers to the technique of irradiating the pattern on the mask by irradiating it with ultraviolet (UV) light, thus forming the designed shape of the mask on the substrate. The general process of photolithography is shown in Figure 3. Photolithography can be further divided into three types: excimer lithography, extreme ultraviolet lithography, and electron beam lithography [43,44,45,46,47,48,49,50,51]. Excimer lithography usually relies on the 193 nm ArF immersion technology. It replaces the air medium between the photoresist and the exposure lens with a liquid medium, which equivalently increases the lens diameter and numerical aperture (NA), thereby improving the resolution. Extreme ultraviolet (EUV) lithography uses EUV light with a wavelength of 10^−14^ nm as the light source, which can reduce the exposure wavelength and expand the lithography technology to feature sizes below 32 nm. According to the Rayleigh formula, extreme ultraviolet light has a very short wavelength for gaining high resolution. Electron beam lithography is an extended application of lithography, which uses a focused electron beam to scan for drawing custom shapes on surfaces coated with electronic resist. The shorter the wavelength of light, the higher the resolution. Because the electron is a wave with an extremely short wavelength, electron beam lithography can process extremely high resolution.

**Figure 4:**
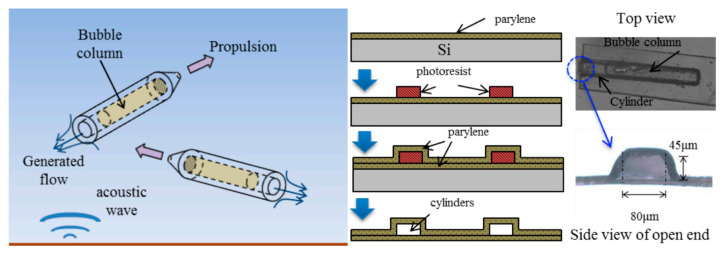
Lithography-fabricated devices for microscale bubble propulsion. Reproduced with permission from Ref. [52].

Since most of the currently developed acoustics-actuated microrobots are still at the micron level, the excimer lithography technology is generally enough to meet the production requirements. Many researchers utilized photolithography to fabricate the acoustics-actuated microrobots (Figure 4) [52]. Typically, the bottom wall of a cylinder is formed by the deposition of a layer of paraxylene on a bare silicon wafer. The photoresist layer is then deposited as the sacrificial layer by the rotating coating on the parylene layer. The side and top walls of the cylinder are then formed by the second layer of paraxylene deposited on top of the sacrificial photoresist layer. Subsequently, reactive ion etching is used to open the tip of the cylinder and separate individual or abreast cylinders from the substrate. Finally, the sacrificial layer is removed by immersing the cylinder in acetone solution. Similarly, by virtue of its simple and straightforward operation, UV polymerization is also demonstrated as a promising tool to manufacture microrobots [53,54].

## 3. Types of Acoustics-Actuated Microrobots

Acoustics-actuated microrobots can be divided into three common types according to their working principles: bubble propulsion, sharp-edge propulsion, and in-situ microrotors. This section aims to articulate key features of these three types of microrobots.

### 3.1. Bubble Propulsion

The working principle of bubble propulsion is due to the vibrations excited by the acoustic field, and the vibration is most intense when the acoustic field frequency reaches the resonance frequency of the bubble. Therefore, as the bubbles vibrate outward and inward, the liquid in the device will be discharged and sucked. Due to the nonlinear terms in the Navier–Stokes equation, this flow pattern generates a source of net momentum, thereby generating a driving force to push the entire device forward. The acoustic waves can provide powerful propulsion forces and acoustically excited bubbles can produce high streaming forces [55,56]. These types of forces on air bubbles in the acoustics field are often called Bjerknes forces [57]. Primary Bjerknes forces are caused by an exterior acoustics field; secondary Bjerknes forces are caused by the interaction between pairs of bubbles in the same acoustics field.

**Figure 5:**
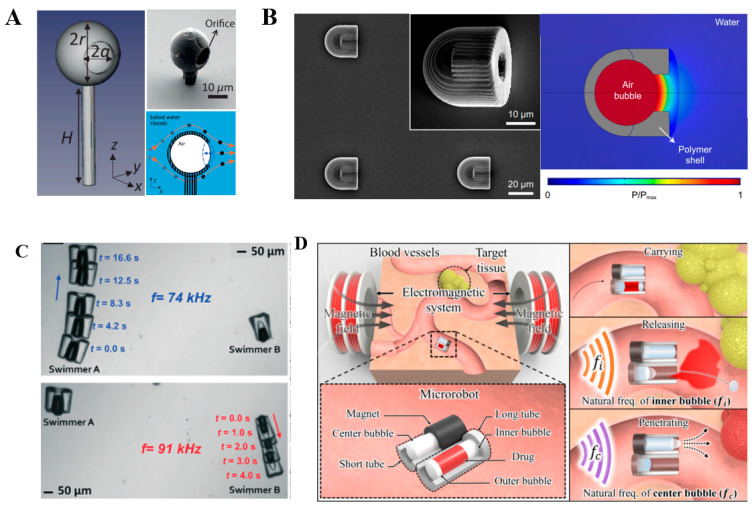
Different designs of bubble propulsion. (**A**) Long-lasting armored microbubble under forced vibration. (**B**) Scanning electron microscopy images of the microrobot. The simulated acoustic pressure map at the resonance frequency. (**C**) The low-power acoustic field actuation oscillatory motion of a bubble enables directional motion in water. Two microswimmers with bubbles of different sizes enable independent control of each body. (**D**) Schematic diagram of proposed drug delivery technology. Reproduced with permission from Refs. [8,59,60,61], respectively.

The principle of bubble propulsion was proposed by Dijkink and co-authors in 2006 [31]. However, convincing verification and analysis were not achieved at the microscale size. With the development of photolithography technology, micro propulsion devices have been manufactured for validating the microscale propulsion mechanism (Figure 4). Upon acoustic actuation of bubble-based microrobot, the propulsion speed and the propulsion force were found to reach as high as 45 mm/s and 6 μN, respectively [52,58]. Two-photon polymerization was also successfully employed to manufacture microrobots. As shown in Figure 5A, an armored microbubble (AMB) was developed with a size of 10–20 μm [59]. Such microbubbles could exist for several hours under forced vibration, providing an alternative route for designing long-lasting robust microrobots. In addition, bubble-actuated microrobots were produced with a streamlined design for making propulsion more efficient [60]. The shape appearance is similar to a bullet (Figure 5B) and the propulsion force could be two to three orders of magnitude stronger than that of natural microorganisms. Given its powerful propulsion force with a propulsion speed of up to 2250 μm/s, such a microrobot holds great promise to overcome the strong blood flow in arteries.

It is generally ineffective for dealing with individual structures because the acoustic field has no selectivity to the object being manipulated. Recently, the application of acoustic radiation fields in a vibratory manner has been introduced to achieve selective addressing. Vibrating bubbles with different sizes trapped inside the microswimmer could generate enough thrust to propel microswimmers and be able to selectively drive individual microswimmers among the group (Figure 5C) [8]. Since the vibration of the bubble is selective to the acoustic field frequency [52], the bubble should vibrate effectively when the frequency of the sound field is equal to the resonant frequency of the bubble. A microrobot platform for drug delivery has been developed based on this principle [61]. The drugs and two bubbles (the inner bubble and the outer bubble, respectively) were arranged in the long tube, and the drugs were wrapped by two bubbles in the long tube. At the specified location, the outer bubble covering the opening of the long tube was discarded and the carried drugs were released by acoustic excitation of the inner bubble (Figure 5D). It is noted that the motion behavior of bubble-propulsion-driven microrobots in low Reynolds flow is complex, especially when the microbubbles are below the size range of 30 μm and vibrate near the solid wall due to nonlinear acoustic forces [59]. These kinds of acoustically driven robots have not been fully studied, and their directional control is still challenging. However, by adding a fin, the microrobot could carry out simple linear movements [60]. In addition, most cylindrical cavity designs still require hydrophobic materials to stabilize bubbles during acoustic actuation.

### 3.2. Sharp-Edge Propulsion

In the bubble propulsion system, bubble propulsion is effective at the resonance frequency of the bubbles. If the bubble bursts, the entire system will lose the driving force. Therefore, the bubble driving system has certain inherent limitations. Alternatively, a sharp-edge propulsion system has been developed to achieve more robust acoustic actuation motions. The sharp-edge propulsion is inspired by single-celled organisms such as *Escherichia coli* and sperm, which move by swinging a sharp-edge-like tail [62,63,64]. Therefore, an artificial sharp-edge structure as the tail of the microrobot was proposed for facilitating the movement of the microrobots under the acoustic field.

When a sharp structure responds to an acoustic drive, its special sharp structure creates a pair of counter-rotating eddies around its tip. This acoustic streaming phenomenon breaks the interface of laminar flow to promote the mass transfer of the fluid. The general theory of acoustic streaming has been widely introduced in the literature [65,66,67]. In addition to producing counter-rotating vortices, the sharp-edge also produces tadpole-tail-like wobbles that create forward propulsion.

**Figure 6:**
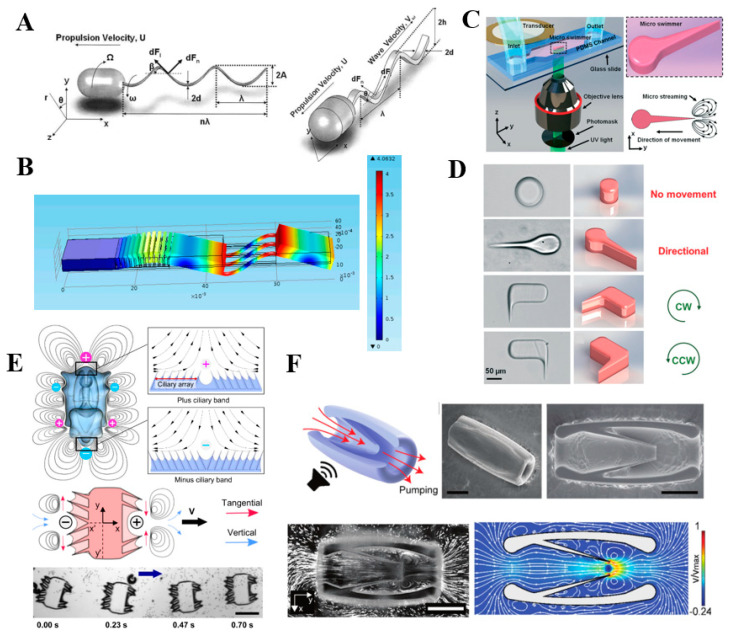
Different structures of sharp-edge propulsion devices. (**A**) Schematic of the microscale swimming robot with helical wave propulsion and the microscale swimming robot with planar wave propulsion. (**B**) COMSOL simulation of the double-articulated tail propulsion. (**C**) Acoustic actuation of the tadpole-like microrobot. (**D**) Different designs of sharp-edge microrobots. (**E**) Starfish-like microrobot. (**F**) Microjet engine based on sharp-edge. Reproduced with permission from Refs. [54,68,69,70,71], respectively.

The concept of sharp-edge propulsion was proposed in 2006 [68]. To date, researchers have developed two different kinds of sharp-edge propulsion methods: helical wave propulsion and planar wave propulsion (Figure 6A). Compared to helical wave propulsion, planar wave prolusion has higher efficiency and performs faster linear velocity. Particularly, double-articulated tail propulsion achieved forward velocities as high as 2.5 mm/s (Figure 6B) [69]. This kind of microrobot exhibited frequency selectivity to realize steering, especially when using multiple flappers with different resonance frequencies. Similarly, a simpler and more straightforward microswimmer was fabricated by photolithography technology (Figure 6C), and the propulsion speed was able to reach up to 1200 μm/s [54]. The movement direction was regulated by changing the sharp-edge structures, and the positions of sharp-edge located on the microrobots could precisely control linear and rotational motion (Figure 6D) [54]. In addition, the propulsion speed was well adjusted by changing the input voltages and the number of sharp-edge structures. Moreover, based on the sharp-edge structures, the research of acoustics-actuated microrobots can be extended to bionic robots [70]. The design of ultrasound-activated synthetic ciliary bands was firstly presented by mimicking starfish cilia. The working principle of cilia is similar to that of sharp-edge; under the action of the acoustic field, the cilia’s vibration would actuate the movement of the starfish-like robot (Figure 6E). Therefore, it is possible to develop a miniature jet engine that transmits fluids through local body deformations without moving parts (Figure 6F) [71], indicating that the microjet engine might be the promising power source of microrobots in the future. Compared to microbubbles, sharp-edge structures possess robust structural stabilities even under extreme working conditions. Therefore, sharp-edge propulsion can be applied in a wider range of situations than bubble propulsion. However, the propulsion speed of sharp-edge propulsion still has extensive room to increase.

### 3.3. In-Situ Microrotor

**Figure 7:**
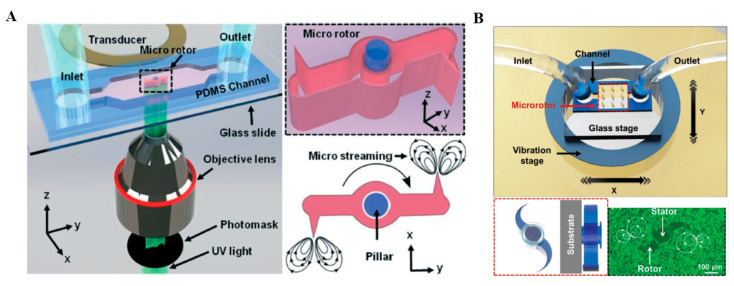
Different structures of in-situ microrotors. (**A**) Acoustic actuation of single in-situ microrotor. (**B**) Acoustic actuation of multiple in-situ microrotors with a microscopic fluorescence image for showing the microstreaming flow lines induced by vibration actuation. Reproduced with permission from Refs. [42,53] respectively.

*In-situ* microrotors are derived from the sharp-edge microrobots with a typically fixed axis. In-situ microrotors can be fabricated using the same manufacturing method as described above (Figure 7A) [53]. As for the fixation of the microrotors, researchers usually design a microcolumn as a shaft in the microchannel. Then, in-situ microrotors are formed upon UV polymerization. They have sharp-edge structures and are asymmetrically positioned on the rotor bar. Piezoelectric transducers are capable of acoustically actuating in-situ microrotors through streaming caused by oscillations of sharp-edge structures. Because these acoustic flows break the reflection symmetry, there is a net torque at the rotor arm. Microstreaming produced by acoustic vibrations could enable an individual 10 μm particle to generate a rotational speed up to 5000 revolutions per minute (RPM) [72]. Inspired by this, a microfluidic platform was developed to control particle motion by adjusting input signals and directional migration [73,74]. Recently, a piezoelectric vibrator has also been proposed to achieve tunable acoustically vibration-induced actuation of multiple in-situ micro-rotors manufactured by two-photon polymerization. (Figure 7B) [42]. The measured rotation speed of the resulting microrotors was able to reach 1600 RPM. Generally, the rotation speed of the in-situ microrotors could be adjusted by the acoustic field frequency and the number of rotor arms. The higher the acoustic field frequency or the greater the number of rotor arms, the faster the rotation speed of the in-situ microrotor. Since these in-situ microrotors are easy to manufacture and actuate, they have promising potential applications in many fields, including micropumps, micromixers, microgear systems, and various microfluidic operations. Table 2 shows the differences among bubble propulsion, sharp-edge propulsion, and in-situ microrotor.

## 4. Applications

With the rapid development of micro-/nanomanufacturing technology, the promising application potential of microrobots has been well demonstrated in the last few decades. Microrobots actuated by acoustics are widely used in many fields due to their unique flexibility and contactless features. This section will highlight the established applications of acoustics-actuated microrobots in targeted drug delivery, microfluidic operation, and microsurgery.

### 4.1. Targeted Drug Delivery

Targeted drug delivery aimed at overcoming the limitations of traditional drugs is an important research direction in the biomedical field. Traditional drugs, in certain situations, cannot reach the treatment site as expected due to the narrowness of blood vessels and weakness of blood flow. Therefore, repeated administration is always employed. However, the increasing toxicity caused by a large quantity of drugs is unavoidable. Alternatively, if microrobots are inserted deep into the human body for targeted drug delivery and to release drugs at designated locations, this can not only minimize side effects, but also reduce the frequency of drug intake and improve the stability of drugs in the body [75].

**Figure 8:**
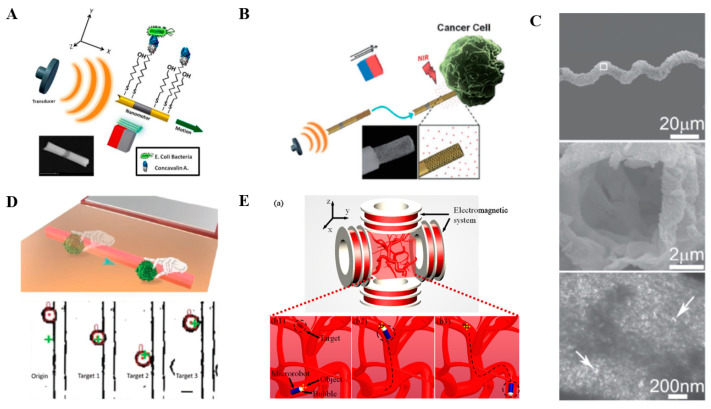
Acoustics-actuated microrobots for targeted drug delivery. (**A**) Ultrasound-propelled magnetically guided nanowire motor for selective capture and transport of biological targets. (**B**) Porous gold nanowire structure for drug loading. (**C**) Structural characterization of the hollow spiral device. (**D**) Precise manipulations of cancer cells based on the vision-feedback control strategy. (**E**) Schematic diagram of micro-object manipulation in blood vessels using a microrobot incorporated with an acoustically oscillating bubble. (**a**) test setup; (**b1**,**b2**,**b3**) micro-object manipulation in human blood vessels using the designed microrobot. Reproduced with permission from Refs. [76,77,78,79,80], respectively.

A nanowire microrobot based on acoustic actuation and magnetic guidance was developed to release drugs in a pH-dependent way [76]. This microrobot was made up of a three-segment Au–Ni–Au nanowire and polypyrrole–polystyrene sulfonate (PPy–PSS) component which was attached to one end of the nanowire (Figure 8A). The drug was positively charged, and after static friction, the drug was loaded onto the negatively charged PPY-PSS component, and the loaded drug was found to be easily released in an acidic environment. Similarly, gold nanowires were developed to be a microrobot with a high drug payload capacity for cancer cell killing (Figure 8B) [77]. In addition, a porous hollow microrobot with a spiral cavity using a biotemplating synthesis method was developed for efficient drug loading. Under the megahertz-level acoustic field, the hollow spiral structure could be destroyed to release the drug (TS2-A-R1, Figure 8C) [78]. Furthermore, a particle swarm was developed as a micromanipulator to transport biological particles (Figure 8D) [79]. Locally enhanced microflows were generated around the micromanipulator, which could generate enough propulsion to propel the particles with different ultrasonic frequencies. Acoustic propulsion can be used not only as a propulsion mechanism for targeted drug delivery but also as a drug-assisted grasping tool. Recently, acoustic vibration bubbles were proposed to manipulate micro-objects (Figure 8E). The propulsion mechanism relies on the electromagnetic actuation, while the acoustic actuation is mainly used as an auxiliary actuation for grasping the device. This kind of microrobot can be operated in a channel of different shapes. By integrating electromagnetic driving and acoustic driving, researchers successfully manipulated fish eggs [80].

### 4.2. Microfluidic Operation

**Figure 9:**
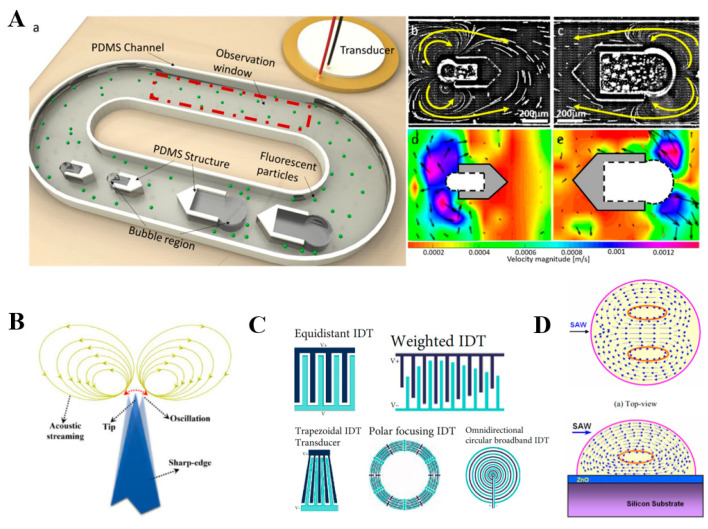
Acoustic-actuated microrobots for microfluidic operation. (**A**) Configuration of the acoustics-based bi-directional micropump device. (**a**) Experimental device structure; (**b**,*c*) acoustic microstreaming flow induced by different sized bubbles; (**d**,**e**) The velocity of particle movement in the microchannel. The actuation voltage is 5 Vpp and the frequencies are 24 kHz and 19 kHz, respectively. (**B**) A typical phenomenon of acoustic streaming produced by a sharp-edge structure in the microfluidic device. (**C**) Different structures of IDT. (**D**) Working schematic diagram of microstirrer. Reproduced with permission from Refs. [81,82,83,87], respectively.

Microfluidic operations can be manipulated well by different kinds of acoustic devices through vibrating bubbles and sharp edges. Volume changes in the vibrating bubble produce a net momentum source so that the fluid in the microchannel flows in a certain direction. By controlling the vibration states of the bubble, a series of specific operations can be performed. For example, acoustic bubbles were utilized to develop a bidirectional micropump [81]. Two kinds of different-sized bubbles were produced to behave at different resonance frequencies. The resonance frequencies of the large bubble and small bubble were found to be 19 kHz and 24 kHz, respectively. When the acoustic field frequency was 19 kHz, the resonation of large bubbles made the fluid flow from the right to the left; when the sound field frequency is 24 kHz, the resonation of small bubbles made the fluid flow from the left to the right (Figure 9A). This micropump was used to carry out the delivery of *E. coli* bacteria, providing promising possibilities for therapeutic drug delivery. Similarly, acoustics can drive the sharp-edge microrobot to rotate, promoting fluid flow. The rotation speed can be well adjusted by changing the acoustic field frequency or the number of rotor arms. When a sharp edge structure is acoustically excited, it oscillates and creates a pair of counter-rotating eddies around its tip (Figure 9B), providing an ideal tool for microfluidic operation [82]. In addition, surface acoustic wave (SAW) devices with different interdigital transducer patterns have been developed for the movement control of small particles [83], precise shunting of microfluidics [84,85,86], and positioning of droplets applications [87]. When the SAW device was utilized as a microstirrer (Figure 9C,D), the stirring speed was well regulated by the acoustic field [88], revealing its superior flexibility.

### 4.3. Microsurgery

Integrating acoustics with other actuation functions (such as magnetics) can create more flexible bioinspired acoustic-magnetic microrobots. Acoustic microrobots have good biocompatibility, while magnetic microrobots have better locomotion and navigation; combining these two actuation modes would have better application prospects in deep tissue.

**Figure 10:**
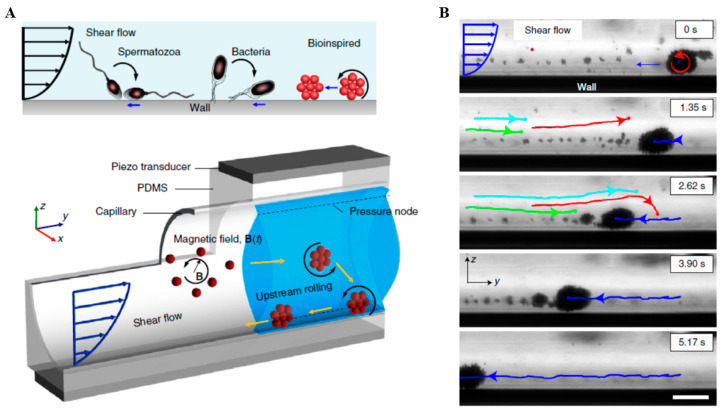
Bioinspired acoustic-magnetic microswarm robots. (**A**) Superparamagnetic particles self-assemble into a spinning microswarm due to the dipole-dipole interaction of a rotating magnetic field. The microswarm was marginalized towards the wall due to the presence of acoustic pressure nodes. (**B**) Image sequences demonstrate the rheotaxis of a microswarm rolling along the wall of the capillary in a combined acoustic and magnetic field. The streamlines of the oppositely directed flow are shown by different colored trajectories. Scale bar = 60 μm. Reproduced with permission from Ref. [90].

Low-frequency ultrasound waves have deep penetration in vivo, so they are often used to help guide the magnetic robots to move. A magnetic spiral micro-robot, driven by a rotating magnetic field and tracked by an ultrasonic system, was developed to clean up blood clots. [89]. After reaching the area to be cleaned, the rotating magnetic field actuated the microrobot to rotate at ω = 35 Hz, rubbing and tearing the fibrin network of the blood clot. Magnetic field and acoustic field could be employed to form a microswarm of particles and to push the micro-group to the wall, respectively, for producing the rolling translational motion (Figure 10) [90]. Similarly, the sperm-templated soft magnetic microrobot was developed by hybrid magnetic-acoustic actuation [91]. The localization of IRON sperms was achieved by ultrasound waves due to the significant improvement in acoustic impedance by iron oxide particles.

In addition to the above, acoustofluidics has been successfully applied to separate micro-/nanoparticles of different sizes. SAW was found to be able to achieve precise screening of microparticles [92] and biological nanoparticles [93] in the microfluidic channels. Besides the separation of microparticles, acoustics was also employed for the enrichment of particles [94]. From both separating and enriching aspects, acoustics holds promising application potential in health monitoring, disease diagnosis, and personalized medicine.

## 5. Current Challenges and Future Perspectives

Given their superior advantages in terms of desirable miniaturization, flexibility, and biocompatibility, acoustics-actuated microrobots have attracted more attention and made remarkable progress during the last decade. However, due to the immature technology, acoustics-actuated microrobots are still in the exploratory stage and many difficulties lie ahead. This section will introduce the main difficulties and the possible future research directions.

From a structural design aspect: (1) The current structural design is still simple. The established microrobots to date can only carry out some basic motions and optimizing the structural design to adapt to different fluidic environments remains very challenging [53,54,90]. (2) There are still very limited material choices available for microrobot fabrication. To broaden their applications in diverse practice settings, a systematic functional materials library should be built for researchers to source from. (3) Photolithography has an obvious flexibility defect for only producing uniform thickness, making it hard for fabricating complicated devices. Although 3D printing is more flexible, the precision is hard to meet the processing requirements.

From a structural application aspect: (1) For bubble actuation, the main difficulty is how to maintain the stability of the bubble. When the bubble is vibrating, its size may change, resulting in a variation in the resonance frequency of the bubble, thereby affecting the effect of propulsion. In more serious cases, the bubble may burst, resulting in the propulsion being ineffective. Therefore, keeping the bubbles at a constant size and working stably is a key point. As described in Section 3.1, AMB (Armored microbubble) could survive for several hours under forced vibration, providing an insightful idea for solving the above-mentioned difficulties [59]. (2) The accurate control of the direction of the robots’ movement is still not achieved [75,76,77]. When the microrobot travels deep into the human body, it is not reliable to rely only on acoustic actuation drugs. Therefore, combining other driving methods such as magnetic driving and electric driving to guide the robot for accurately targeting the desired locations is very necessary. Moreover, an instant feedback system should be built to be able to transmit information outside on time. (3) How to retrograde microrobots in fluids is another difficult problem [90]. A possible solution is shown in Figure 10. However, when the liquid flow rate is fast, those micro-swarm robots could no longer continue to travel retrogradely.

Acoustics-actuated microrobots have attracted considerable attention in recent years, especially the tiny dimensions and flexible motions that allow them to access small, confined, and deep body parts that conventional medical devices cannot reach [95]. The acoustic field is a reliable actuating source, which can remotely and controllably actuate the movement of the microrobots. As an emerging technology, acoustics-actuation is an innovative and promising manner for contactless propulsion and the control of microrobots. Although the technology of acoustics-actuation is still not mature enough, it shows significant potential and will certainly benefit diverse research fields, such as treating arteriosclerosis, removing blood clots, cleaning wounds, and removing parasites. Regarding the challenging problems mentioned above, some researchers have already provided certain insightful solutions. It is believed that as manufacturing technology develops, acoustics-actuated microrobots will play vital roles and change our lives.

## 6. Conclusions

This review provided a comprehensive overview of the research and development of acoustics-actuated microrobots. Specifically, we introduced the manufacturing methods (3D printing and photolithography), summarized different microrobot types (bubble prolusion, sharp-edge propulsion, and in-situ microrotor) based on their working principles, and discussed the established applications of acoustics-actuated microrobots (targeted drug delivery, microfluidic operation, and microsurgery). Although acoustics-actuated microrobots are still in the early stage and many challenges lie ahead, the booming development of microscale manufacturing technologies will significantly advance their functions and structures towards practical engineering and scientific applications.

## Figures and Tables

**Table 1:** Comparison of different actuation methods.

Actuation Method	Advantages	Major Limitations
Magnetic actuation	Biocompatible power supply; Relatively reliable operability; Strong penetration; Long action time	Microrobots are difficult to fabricate because of their special shape; Difficulty in selective microrobot control
Biological actuation	Biocompatible power supply; Combination of the actuating and sensing capabilities; Natural suitability for physiological fluids; Relatively high efficiency	Needs of a particular environment containing proper nutrients for fueling the microrobot
Chemical actuation	Fast actuation speed; Use of biocompatible fuel such as urea, glucose, H_2_O, and acids in self-phoretic propulsion	High risk of in vivo cross-reactivity; Insufficient propulsion accuracy; Short action time; Lack of instantaneous feedback
Acoustic actuation	Biocompatible power supply; Capability to control microrobot deep inside of the body; Strong penetration; High flexibility; Low power consumption; Long action time	Requirements of proper instrumentation for in vivo use; The material and design requirements of microrobots are relatively strict

**Table 2:** The differences among bubble propulsion, sharp-edge propulsion, and in-situ microrotor.

Propulsion Type	Principle	Advantages	Disadvantages
Bubble propulsion	Bubbles’ vibration produces a source of net momentum	Fast and strong propulsion	Narrow frequency selection range; Bubbles may burst; Hard operation
Sharp-edge propulsion	Sharp-edge’s vibration generates propulsion	Wide frequency selection range; Simple manufacturing, and operation	Low propulsion
In-situ microrotor	Similar to sharp-edge propulsion, but there is a fixed axis in the center	In-situ propulsion; Low fluid influence	Complex design
